# Space Allowance of the Littered Area Affects Lying Behavior in Group-Housed Horses

**DOI:** 10.3389/fvets.2017.00023

**Published:** 2017-03-07

**Authors:** Joan-Bryce Burla, Christina Rufener, Iris Bachmann, Lorenz Gygax, Antonia Patt, Edna Hillmann

**Affiliations:** ^1^Ethology and Animal Welfare Unit, ETH Zurich, Zurich, Switzerland; ^2^Agroscope, Swiss National Stud Farm, Avenches, Switzerland; ^3^Centre for Proper Housing of Ruminants and Pigs, Federal Food Safety and Veterinary Office FSVO, Bern, Switzerland

**Keywords:** horse, lying behavior, duration of recumbency, rapid eye movement sleep deficiency, social behavior, rank, housing conditions, welfare

## Abstract

Horses can sleep while standing; however, recumbency is required for rapid eye movement (REM) sleep and therefore essential. Previous research indicated a minimal duration of recumbency of 30 min per 24 h to perform a minimal duration of REM sleep. For group-housed horses, suitable lying area represents a potentially limited resource. In Switzerland, minimal dimensions for the space allowance of the littered area are therefore legally required. To assess the effect of different space allowances of the littered area on lying behavior, 38 horses in 8 groups were exposed to 4 treatments for 11 days each; T0: no litter provided, T0.5: 0.5× minimal dimensions, T1: minimal dimensions, and T1.5: 1.5× minimal dimensions. Non-littered areas were covered with hard rubber mats. Lying behavior was observed during the last 72 h of each treatment. The total number of lying bouts per 24 h was similar in treatments providing litter, whereas in treatment T0, recumbency occurred only rarely (*F*_1,93_ = 14.74, *p* = 0.0002) with the majority of horses lying down for less than 30 min per 24 h ($\chi_{1}^{2}=11.82$, *p* = 0.0006). Overall, the total duration of recumbency per 24 h increased with increasing dimensions of the littered area, whereby the effect attenuated between treatment T1 and T1.5 in high-ranking horses but continued in low-ranking horses (*F*_1,91_ = 3.22, *p* = 0.076). Furthermore, low-ranking horses showed considerably more forcedly terminated lying bouts in treatments T0.5 and T1, but were similar to high-ranking horses in T1.5 (*F*_1,76_ = 8.43, *p* = 0.005). Nonetheless, a number of individuals showed durations of recumbency of less than 30 min per 24 h even in treatment T1.5. The lying behavior was dependent on the availability of a soft and deformable surface for recumbency. A beneficial effect of enlarged dimensions of the littered area was shown by increased durations of recumbency and decreased proportion of forcedly terminated lying bouts in low-ranking horses. Taking this into account, it became evident that the minimal dimensions for the littered area as implemented in the Swiss animal welfare legislation do not ensure undisturbed lying behavior for all members of a given group.

## Introduction

Horses invest the majority of their time-budget in feeding and resting behavior (1–3). Resting behavior, which includes periods of inactivity and sleep, occupies 5–7 h of the day (4–6). Horses show a polyphasic pattern of resting with the total duration distributed to multiple shorter periods. Sleep occurs in some of these resting periods (6), where the majority of sleep takes place during the dark period after midnight (2, 7, 8).

Sleep is one of the most relevant behaviors for biological functioning (9, 10). It has been studied in many species (11) but only a small number of studies have been conducted in horses. In contrast to most other species, sleep in horses is not uniquely associated with recumbency as horses are able to go through some sleep stages while standing (2, 12). Accordingly, sleep does not necessarily implicate recumbency. On the other hand, inferring sleep from recumbency is quite reliable as horses usually fall asleep shortly after lying down (2); what may be an adaptive response for minimizing time spent in a vulnerable position (12). Wakefulness, drowsiness, slow-wave-sleep, and paradoxical or rapid eye movement (REM) sleep are the most frequently mentioned stages for horses’ sleep–wake rhythm; but no consistent terminology exists (13). These stages do not necessarily correspond to the positions a horse adopts (8, 12–15). Wakefulness, drowsiness, and short-wave-sleep can occur in every position, i.e., while standing or during sternal and lateral recumbency. REM sleep is the only sleep stage for which recumbency—sternal with muzzle resting on the ground or lateral (16)—is required due to the total loss of muscle tone in facial, postural, and respiratory (except for the diaphragm) muscles (17).

As different aspects of the function of sleep are accomplished in the different stages, all stages are necessary for physiological and psychological recovery (9, 18). Taking this into account, REM sleep and as a consequence recumbency is essential for horses. If horses are prevented from lying down, the duration of short-wave-sleep may increase but when they are able to adopt recumbency again, a rebound in REM sleep with increased durations occurs the following two or three nights (2). Nonetheless, horses are sensitive with regard to the conditions under which they lie down. Unsuitable environmental conditions or social insecurity but also physical complaints are reasons for which horses may be reluctant to lie down (4–6, 8, 19–21). Although it is mentioned that horses can tolerate more than 3 months without recumbency (unpublished, cited by 22), REM sleep deficiency due to recumbent sleep deprivation has not been investigated in detail yet. Only few case studies addressing REM sleep deficiency in horses exist (19, 23, 24). All authors describe symptoms of excessive drowsiness in horses which were reluctant to occupy a recumbent position, as these horses transition into REM sleep while standing, partially collapse, and then wake up suddenly (12); a behavior which is commonly but incorrectly diagnosed as narcolepsy (23, 25).

However, a small number of studies measured REM sleep duration in horses with presumably normal resting behavior. Dallaire and Ruckebusch measured average REM sleep durations of 41.7–52.8 min/night in four ponies during four nights (7) and 31.6–53.68 min/night in three ponies during three nights (26), Dallaire (2) reports 57.6 min/night in five individually stabled ponies and 28.8 min/night in two ponies in a paddock, and Kalus (13) measured 22.5–37.2 min/night in seven horses during four nights. Furthermore, all studies showed small inter- and intra-individual differences in daily REM sleep durations [e.g., Kalus (13): $\bar{x}$ ± SD: 31.3 ± 9.9 min/night]. Derived from these findings, it can be assumed that horses need a minimal duration of recumbency of 30 min per 24 h to perform a minimal duration of REM sleep (12, 13, 27).

In accordance therewith, feral and semi-wild horses were observed to spend 30 min up to 2.7 h recumbent per day (3, 8, 28–30), preferring a dry, clean, and soft surface in an open space for recumbency (8, 21, 31, 32). Seasonal variations occur as changes in weather influence ambient conditions (5, 8, 33). In addition, age and gender are factors causing differences in the duration of recumbency (3, 29, 34). Foals and juvenile horses spend considerably more time recumbent, particularly in lateral position, than adult horses. Females with foals are lying less than adult males (34). Rank status of an individual, on the other hand, is rather insignificant since the lack of spatial limitations under natural conditions seems to allow each individual within a group to satisfy its demand for recumbency (32, 33).

Under housing conditions, multiple factors were found to affect lying behavior in stabled horses. Whereas little is known about gender (13), age was found to have a similar effect as in feral horses (35–37). Regarding the location for recumbency, areas with litter, i.e., a soft and deformable surface, are preferred compared to non-littered areas (38). The space allowance of the littered area affects lying behavior, specifically in group-housed horses (35, 37). Regarding the impact of group size, contradictory observations have been made and it remains unclear whether horses in smaller or larger groups show more recumbency (35, 36, 39). Further, low-ranking horses often show decreased durations of recumbency compared to high-ranking horses (35, 37, 40). Consequently, in order to ensure undisturbed lying behavior for all members of a given group, the provision of sufficiently suitable lying area, with respect to space allowance and comfort, is a welfare issue which has to be taken into account (8, 41, 42).

For this reason, requirements for the minimal dimensions of the littered area of group housing systems (dependent on the withers height of the individual group members) have been implemented in the animal welfare legislation in Switzerland (43, 44). However, these minimal dimensions have developed historically and are not based on scientific evidence, as experimental studies investigating lying behavior in group-housed horses under systematically varied conditions are not available. Therefore, the aim of this study was to investigate how the space allowance of the littered area affects the lying behavior and, furthermore, to examine the adequacy of the legal requirements for the littered area of horses housed in multi-roomed group housing systems. Thereby, increased numbers of lying bouts as well as increased durations of sternal and lateral recumbency were expected with increasing space allowances of the littered area. Moreover, lying behavior was expected to differ between low- and high-ranking horses, specifically with regard to forcedly terminated lying bouts.

## Materials and Methods

### Horses, Groups, and Group Housing Systems

The study was conducted from March to June 2014 with 38 horses housed in 8 groups on 6 different farms in Switzerland. The groups consisted of three to seven horses (Table 1) aged between 1 and 22 years $\text{(}\bar{x}\pm \text{SD: 11.5}\pm \text{5.4 years)}$. The withers height of the individuals measured 70–170 cm and included 22 ponies (≤148 cm) and 16 large horses (>148 cm; Table 1). The sex ratio within the groups varied (1 group with geldings only, 4 with mares only, 3 mixed) and resulted in a total of 29 mares and 9 geldings. Most horses were privately owned and used as leisure riding horses, riding school ponies, or not used (26 individuals in 6 groups), whereas some horses were used in medical research on reproduction or embryo transfer (12 horses in 2 groups).

**Table 1:** **Farm affiliation, number of horses per withers height category, and calculated dimensions of the littered area per treatment for each group**.

Farm	Group	No. of horses per withers height category (cm) and corresponding minimal dimensions^a^						Dimensions of littered area (m^2^)			

		<120	120–134	134–148	148–162	162–175	>175	T0	T0.5	T1	T1.5
	
		4.0 m^2^	4.5 m^2^	5.5 m^2^	6.0 m^2^	7.5 m^2^	8.0 m^2^				
A	1	4	0	0	0	0	0	0.0	8.0	16.0^b^	24.0
A	2	3	1	0	0	0	0	0.0	8.3	16.5^b^	24.8
B	3	7	0	0	0	0	0	0.0	14.0	28.0^b^	42.0
C	4	1	0	0	3	0	0	0.0	11.0	22.0	33.0^b^
D	5	0	0	0	1	5	0	0.0	21.8	43.5	65.3^b^
D	6	0	0	0	1	5	0	0.0	21.8	43.5	65.3^b^
E	7	1	0	1	1	0	0	0.0	7.8	15.5	23.3^b^
F	8	1	0	3	0	0	0	0.0	10.2	20.5^b^	30.8

*^a^FSVO (43)*.

*^b^Status quo of the space allowance of the littered area before participation in the study*.

All horses were group-housed day and night in multi-roomed group housing systems with at least two spatially separated areas, i.e., an outdoor run and an indoor area. Requirements for participating in the study were as follows:

- A general compliance of the Swiss animal welfare legislation for horses (43, 44).
- The indoor area allowed ≥1.5× the legally required minimal dimensions (= experimental area) for the littered area of multi-roomed group housing systems (Table 1).
- Litter materials used in lying areas were straw or wood shavings.
- The ground of the outdoor run was firm, i.e., paved ground (in order to make recumbency unappealing in other areas than the experimental area).

*Status quo* of the space allowance of the littered area before participation in the study corresponded to either the minimal dimensions (in four of the eight groups) or 1.5× the minimal dimensions (in the other four groups; Table 1). The lying areas had no structural elements but in two groups the experimental areas were divided into two separate areas by solid walls. During the study, the experimental area was covered with hard rubber mats (vulcanized rubber, thickness approximately 2 cm; e.g., Gummimatte Standard, StarParade GmbH, Benken, Switzerland).

Customary stable management was practiced on all farms and was not changed for the study. Feeding of the horses took place outside of the lying area, either in the outdoor run or in an additional feeding area. The feeding management differed greatly between groups; some groups were fed *ad libitum* with hay and straw, others were fed rationed either with hay or with both hay and straw. In order to make recumbency on pasture less appealing, groups were allowed access to pasture for a maximum of 4 h per day. In order to identify lying bouts on pasture, the farm owners were required to take daily notes on the beginning and end of pasture access.

### Experimental Conditions

The legally required minimal dimensions for the littered area of multi-roomed group housing systems depend on the withers height of the individual group members (43, 44) which are summed to calculate the minimal dimensions for a particular group. Accordingly, the absolute dimensions of the littered area differed for each group (Table 1).

Each group was exposed to each of the following four treatments with different space allowances of the littered area; designations of treatments refer to the ratio of the legally required minimal dimensions for the littered area (Table 1):

- T0: no litter, 1.5× minimal dimensions covered with rubber mats. (Horses were provided with a hydroscopic surface sufficient for species-appropriate staling but not suitable for recumbency. Groups accustomed to straw as litter material were provided with straw for forage supply in a fodder rack.)
- T0.5: 0.5× minimal dimensions littered + minimal dimensions covered with rubber mats.
- T1: minimal dimensions littered + 0.5× minimal dimensions covered with rubber mats.
- T1.5: 1.5× minimal dimensions littered, no (uncovered) rubber mats.

Each treatment included 8 days of habituation and 3 days (72 h) of continuous data recording, followed by one transition day to adapt the littered area according to the following treatment. The order in which the four treatments were applied in the different groups was systematically balanced; three groups started with their *status quo* (Table 1), no group started with treatment T0 (to avoid an initial extreme condition), and not more than two groups had the same treatment simultaneously.

### Data Recording and Processing

#### Accelerometer

**Figure 1:**
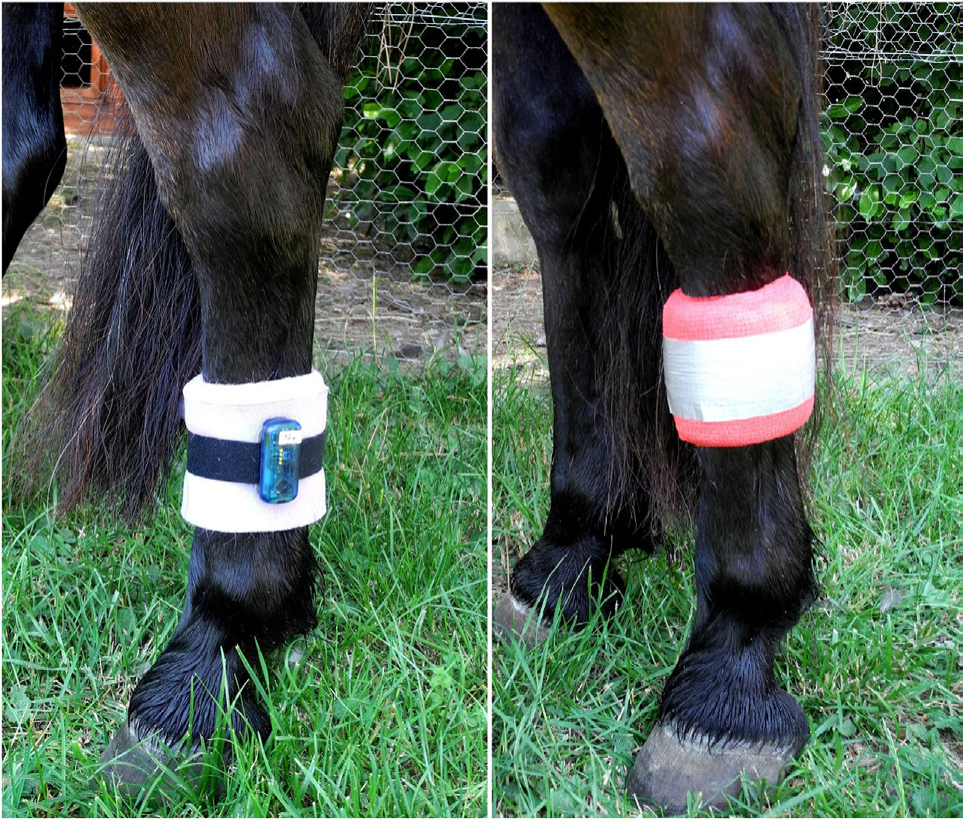
**Accelerometer attached to the left hind leg with a Velcro strap and foam material underneath (left) and protected from damage with foam material, elastic bandages, and duct tape (right)**.

During the periods of data collection (72 h per treatment), the lying behavior of each horse was recorded automatically using an accelerometer of the type MSR145 data logger (MSR Electronics GmbH, Seuzach, Switzerland). The devices were attached to the metacarpal bone of the left hind leg with a Velcro strap and foam material was used underneath to prevent pressure to the leg (Figure 1). Further, foam material, elastic bandages, and duct tape were used to protect the devices from damage (Figure 1). The accelerometers recorded the acceleration parallel to the vertical leg movement with a frequency of 1 Hz. The application is based on the principle of a lack of gravitational force in horizontal position, i.e., during horizontal position of the animals’ metacarpal bone in sternal as well as lateral recumbency, resulting in acceleration values close to 0 during recumbency. By contrast, standing and locomotion result in varying acceleration values (45). The accelerometer has previously been used to record lying behavior in cows (46–48) and goats (49). Additionally, the reliability of our data was validated by comparing the indicated lying bouts of three randomly chosen horses during the first 24 h of data collection with continuously recorded video footage.

MSR data were transferred to a computer *via* MSR Software (version 5.28.07) and saved as CSV-files. R [version 3.0.3; (50)] was used for automatic detection of lying bouts by means of registering a change in position (vertical/horizontal) as indicated by a change in acceleration values. Every single data point was assigned to either lying if the acceleration was greater than −0.75 g or standing if the acceleration was smaller than −0.75 g. To account for fluctuations caused by locomotion, a moving median was then smoothed across 30 s and data points were set as either lying or standing if a majority of the points in the 15 s before and 15 s after the data point in question indicated one or the other state, respectively. The output included the exact start and end time as well as duration of each lying bout. Lying bouts were considered for analysis if they had a minimum duration of 30 s. Based thereon, the number of lying bouts per 24 h and the duration of recumbency per 24 h were determined for each horse, resulting in three data points (3 × 24 h) per horse per treatment. For each 24 h period, it was further assessed whether the duration of recumbency of an individual was less than 30 min.

#### Video Recording

**Figure 2:**
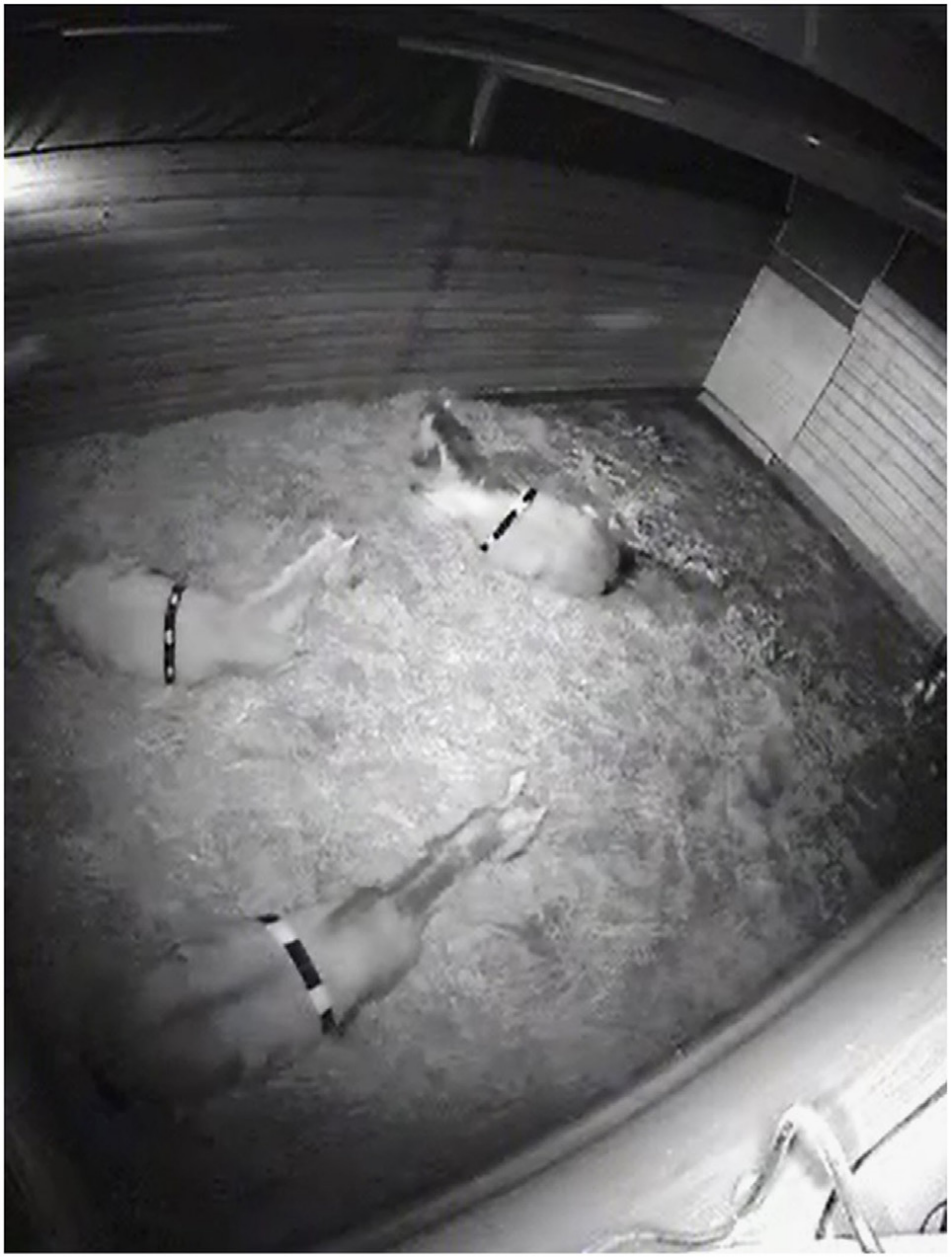
**Video footage of the indoor area**. For individual identification, horses were equipped with elastic belts (infrared light absorbing) with different color patterns (infrared light reflecting).

Continuous video footage was recorded over 72 h by using multiple infrared video cameras per group to guarantee complete surveillance of the entire indoor area. To distinguish individuals on the infrared video footage, the horses were equipped with elastic belts (infrared light absorbing) marked with different patterns of infrared reflecting color (Streicolor AG, Frauenfeld, Switzerland; Figure 2). Based on information on start and end time of each lying bout supplied by MSR data, the following variables were observed (by one researcher based on a predefined protocol) for each lying bout:

- Location: possible locations for recumbency within the experimental area were either litter or rubber mats. In group housing systems with an indoor area larger than the experimental area (>1.5× minimal dimensions), recumbency was also possible on concrete. Further, if a lying bout was listed in the MSR data output but the horse was not visible on the video footage of the indoor area, it was assumed that recumbency took place in the outdoor run. Therefore, lying bouts that took place in locations other than litter or rubber mats were logged as recumbency on firm ground.
  Lying bouts on pasture were very rare and only of short duration (in total 14 lying bouts of <1 min and 5 lying bouts of 1–8 min), wherefore recumbency on pasture was neglected in the further data analysis.
- Duration of sternal and lateral recumbency: the behavioral observation software INTERACT^®^ (Mangold International GmbH, Arnstorf, Germany) was used to determine the duration of recumbency in sternal and lateral position [according to Pedersen et al. (16)].
  – Sternal recumbency: “The asymmetrical sterno-abdominal posture in which the lateral surfaces of the flexed underneath limbs are applied to the ground in such a way that the sternum and abdomen rest on the ground to the right or left of the midline, with the muzzle resting on the ground, a forelimb, or not at all.”
  – Lateral recumbency: “A right or left posture in which the upper forelimb is anterior to the lower forelimb, the hind limbs are extended, and the head is resting on the ground.”
  The proportion of lateral recumbency in relation to the total duration of recumbency per 24 h was calculated for each horse per 24 h.
- Group members present when lying down: the number of horses present in the experimental area at the beginning of each lying bout (moment when abdomen of the individual touched the ground) was counted. The average proportion of group members present in relation to the total number of group members was calculated for each horse per 24 h.
- Termination of lying bouts: the cause for the termination of a lying bout was categorized as either self-determined or forced (as a direct response to the action of another horse, e.g., displacement, threatening, or aggressive behavior). The average proportion of forcedly terminated lying bouts in relation to the total number of lying bouts was calculated for each horse per 24 h.

#### Rank Tests

Paired feeding tests were conducted in each group to determine the rank status of each individual (51–53). Prior, all horses were introduced individually for 2 min to a bucket with concentrate feed which was used to cause competition during the paired encounters. When conducting the feeding test, each possible dyad of group members was tested in a random order; each individual was only tested in two consecutive encounters to allow the horses to recover from potential stress. Each encounter took no longer than 3 min, which was given by the relatively small amount of concentrate feed (approximately 100 g) presented in the bucket. The dominant individual of each pair was determined with regard to agonistic behavior, displacement, and time spent feeding.

Based thereon, the ratio of the number of dominated dyads to the total number of dyads was calculated for each horse according to Sambraus (54) and horses were assigned to be either “low-ranking” (0–0.5) or “high-ranking” (>0.5–1).

### Statistical Analysis

Statistical analysis was conducted in R [version 3.2.1; (50)] using linear mixed-effects models [lme, package “nlme”; (55)] and generalized linear mixed-effects models [glmer, package “lme4”; (56)]. Model assumptions were checked using graphical analysis of residuals focusing on normality of errors and random effects, homoscedasticity of the errors in case of normally distributed errors, and absence of bias in mean errors for generalized models; outcome variables were transformed if necessary (Table 2). The final models were obtained by a stepwise backwards reduction with a *p*-value of >0.1 as the criterion of exclusion.

**Table 2:** **Overview of outcome variables and analyzed locations with respective calculated model type and transformation (if necessary)**.

Outcome variable	Analyzed locations	Model type (transformation)
Number of lying bouts (*n* per 24 h)	Total On litter On rubber mats On firm ground	lme lme lme lme
Duration of recumbency (min per 24 h)	Total On litter On rubber mats On firm ground	lme lme lme (log) lme (log)
Duration of recumbency <30 min per 24 h (yes, no)	Total On litter	glmer glmer
Lateral recumbency (%)	Total	lme (logit)
Group members present when lying down (%)	Total	lme
Forcedly terminated lying bouts (%)	Total	lme

Outcome variables recorded for each horse per 24 h included the number of lying bouts, the duration of recumbency, whether duration of recumbency was less than 30 min per 24 h, the proportion of lateral recumbency, the proportion of group members present in the experimental area when lying down, and the proportion of forcedly terminated lying bouts (Table 2). In order to assess recumbency in different locations, certain outcome variables were additionally analyzed separately for recumbency on litter (provided in treatments T0.5, T1, T1.5), on rubber mats (provided in treatments T0, T0.5, T1), and on firm ground (Table 2). Fixed effects included in the full models were treatment (ordered factor), rank status (factor with two levels: low-ranking, high-ranking), and their interaction. The ordered factor for treatment was coded as a third-order polynomial. This allowed the reduction of the model to a second-order polynomial or a linear relationship between treatment and the outcome variables. Accordingly, we can report the most parsimonious description of this relationship. For outcome variables analyzed separately for different locations, the ordered factor for treatment was coded as a second-order polynomial only. The random effect included the experimental condition nested in the individual horse nested in the group.

A total of 19 horse/treatment combinations (12.5%) had to be excluded from the analysis due to either one of the following reasons: mild symptoms of acute laminitis (1 horse in T0), medical treatment for the purpose of research (1 horse in T0, 2 horses in T0.5, 4 horses in T1, and 3 horses in T1.5), or being fed with hay under suspicion of containing the poisonous plant *Colchicum autumnale* (4 horses in T0 and 4 horses in T1).

## Results

### Number of Lying Bouts

**Figure 3:**
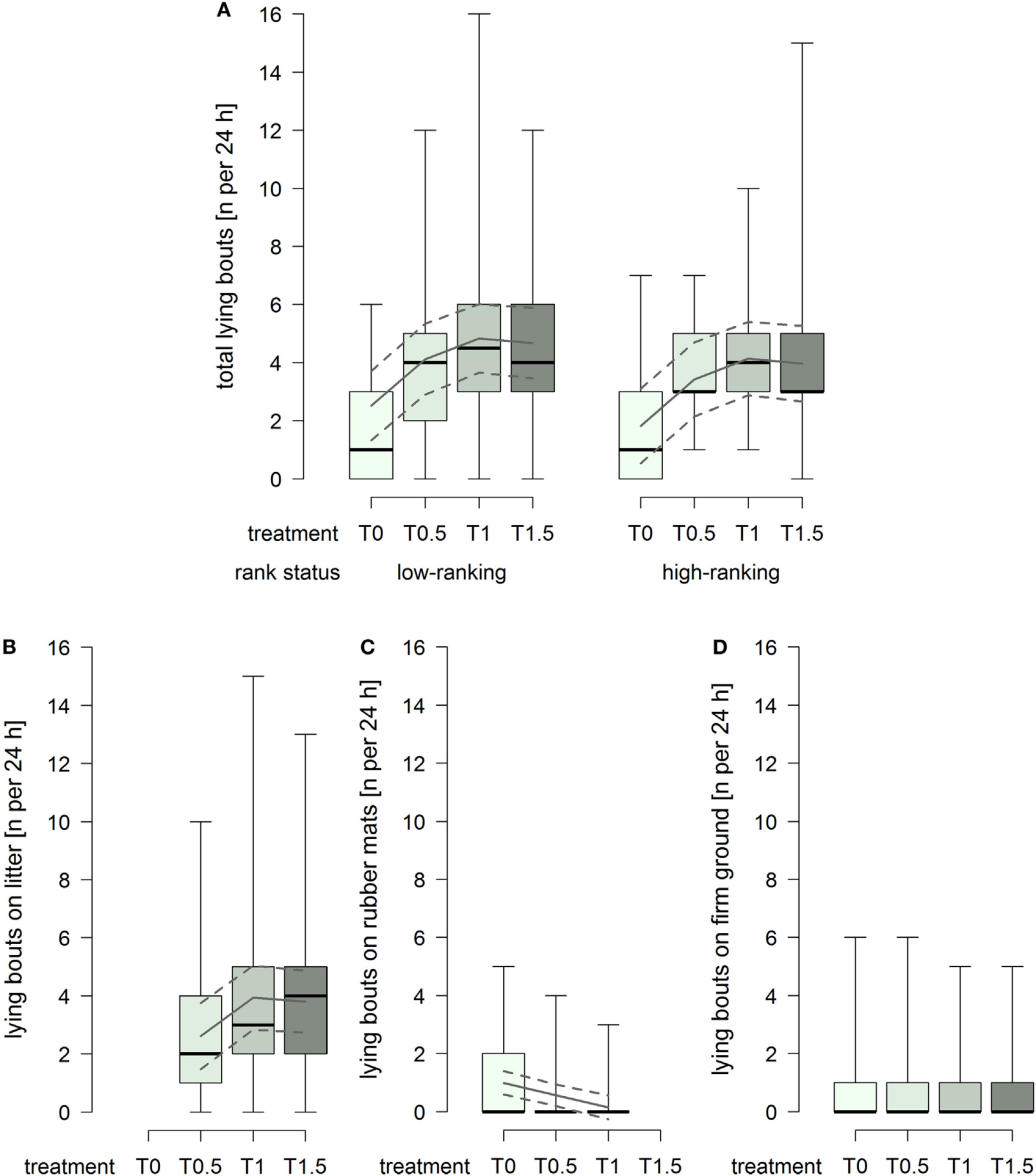
**Number of lying bouts per individual per 24 h in treatments T0, T0.5, T1, and T1.5 for (A) total (recumbency on litter, rubber mats, and firm ground), (B) on litter, (C) on rubber mats, and (D) on firm ground**. Boxplots show medians, interquartiles, and absolute ranges of data. In addition, model estimates (solid lines) and 95% confidence intervals (dashed lines) are shown.

The total number of lying bouts was lower in treatment T0 compared to the treatments providing litter (treatment^2^: *F*_1,93_ = 14.74, *p* = 0.0002; Figure 3A), and low-ranking horses generally showed a higher total number lying bouts than high-ranking horses (rank status: *F*_1,29_ = 3.25, *p* = 0.082; Figure 3A). The number of lying bouts on litter increased with increasing dimensions of the littered area, whereby the effect attenuated between treatment T1 and T1.5 (treatment^2^: *F*_1,61_ = 4.91, *p* = 0.031; Figure 3B). The number of lying bouts on rubber mats was generally low and decreased continuously with increasing dimensions of the littered area, approaching 0 in treatment T1 (treatment: *F*_1,59_ = 20.12, *p* < 0.0001; Figure 3C), and low-ranking horses [model estimate (95% confidence intervals): 0.70 (0.27, 1.12)] showed a higher number lying bouts on rubber mats than high-ranking horses [0.39 (−0.07, 0.86)] (rank status: *F*_1,29_ = 4.43, *p* = 0.044). The number of lying bouts on firm ground was constantly low and an effect of treatment or rank status was not evident (Figure 3D).

### Duration of Recumbency

**Figure 4:**
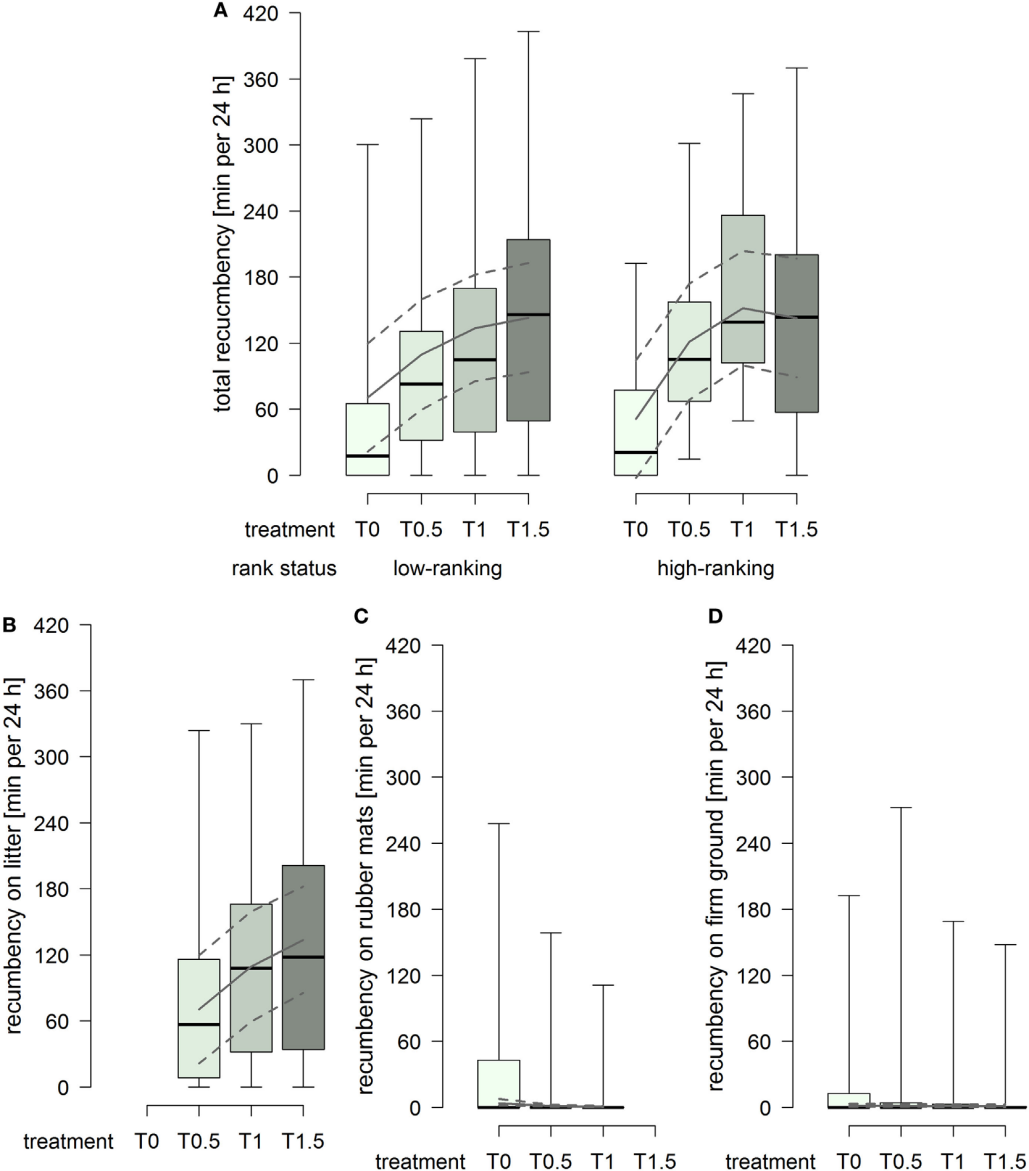
**Duration of recumbency per individual in minutes per 24 h in treatments T0, T0.5, T1, and T1.5 for (A) total (recumbency on litter, rubber mats, and firm ground), (B) on litter, (C) on rubber mats, and (D) on firm ground**. Boxplots show medians, interquartiles, and absolute ranges of data. In addition, model estimates (solid lines) and 95% confidence intervals (dashed lines) are shown.

The total duration of recumbency increased with increasing dimensions of the littered area, whereby the effect attenuated between treatment T1 and T1.5 in high-ranking horses but continued in low-ranking horses (treatment^2^ × rank status: *F*_1,91_ = 3.22, *p* = 0.076; Figure 4A). The duration of recumbency on litter increased continuously with increasing dimensions of the littered area (treatment^2^: *F*_1,61_ = 3.61, *p* = 0.062; Figure 4B). The duration of recumbency on rubber mats was generally low and decreased with increasing dimensions of the littered area, approaching 0 already in treatment T0.5 (treatment^2^: *F*_1,93_ = 3.01, *p* = 0.086; Figure 4C). The duration of recumbency on firm ground was generally low and decreased with increasing dimensions of the littered area (treatment: *F*_1,94_ = 4.09, *p* = 0.046; Figure 4D).

**Figure 5:**
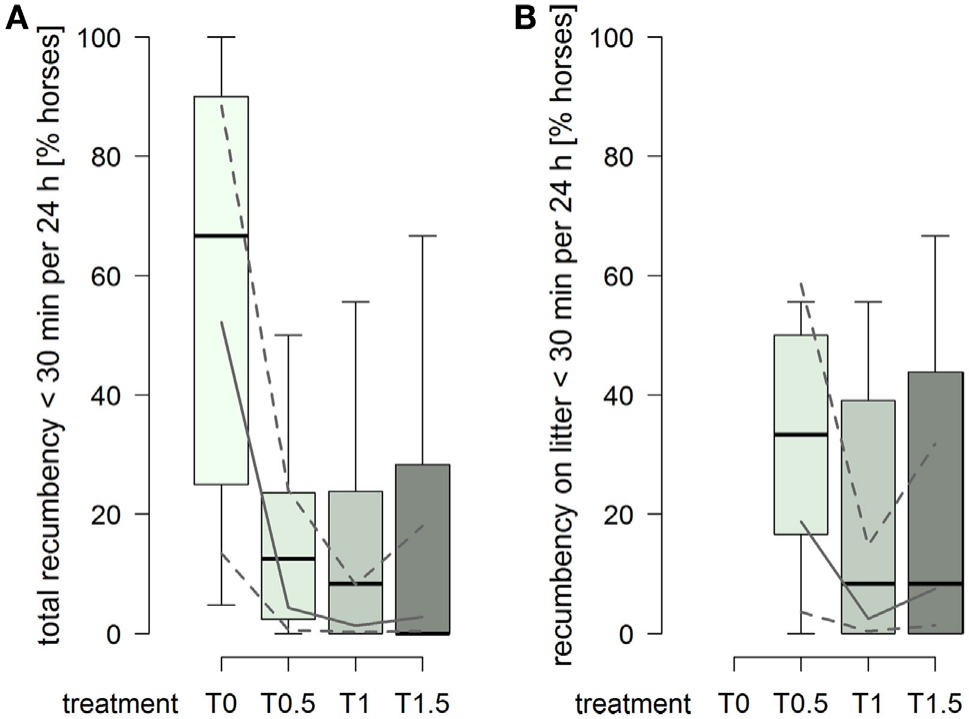
**Proportion of horses per group with durations of recumbency of less than 30 min per 24 h in treatments T0, T0.5, T1, and T1.5 for (A) total (recumbency on litter, rubber mats, and firm ground) and (B) on litter**. Boxplots show medians, interquartiles, and absolute ranges of data on group level. In addition, model estimates (solid lines) and 95% confidence intervals (dashed lines) are shown.

The probability for a horse to have a total duration of recumbency of less than 30 min per 24 h was higher in treatment T0 compared to treatments providing litter (treatment^2^: $\chi_{1}^{2}=11.82$, *p* = 0.0006; Figure 5A). Furthermore, 15 horses were also recumbent for less than 30 min over the entire 72 h of data recording in T0, compared to 1 horse in T0.5 and T1, and 2 horses in T1.5. For recumbency on litter, the probability for a horse to have a duration of recumbency of less than 30 min per 24 h was clearly higher in treatment T0.5 compared to treatments T1 and T1.5 (treatment^2^: $\chi_{1}^{2}=6.29$, *p* = 0.012; Figure 5B).

### Lateral Recumbency

The proportion of lateral recumbency increased continuously with increasing dimensions of the littered area from 0.67% (0.18%, 2.4%) in treatment T0 to 1.04% (0.3%, 3.55%) in T0.5, 1.63% (0.49%, 5.28%) in T1, and 2.54% (0.74%, 8.37%) in T1.5 (treatment: *F*_1,79_ = 10.05, *p* = 0.002).

### Group Members Present when Lying Down

The proportion of group members present in the experimental area at the moment of lying down increased continuously with increasing dimensions of the littered area from 52.42% (39.1%, 65.73%) in treatment T0 to 55.21% (41.92%, 68.5%) in T0.5, 58.0% (45.0%, 71.0%) in T1, and 60.79% (47.59%, 74.0%) in T1.5 (treatment: *F*_1,79_ = 6.63, *p* = 0.012).

### Termination of Lying Bouts

**Figure 6:**
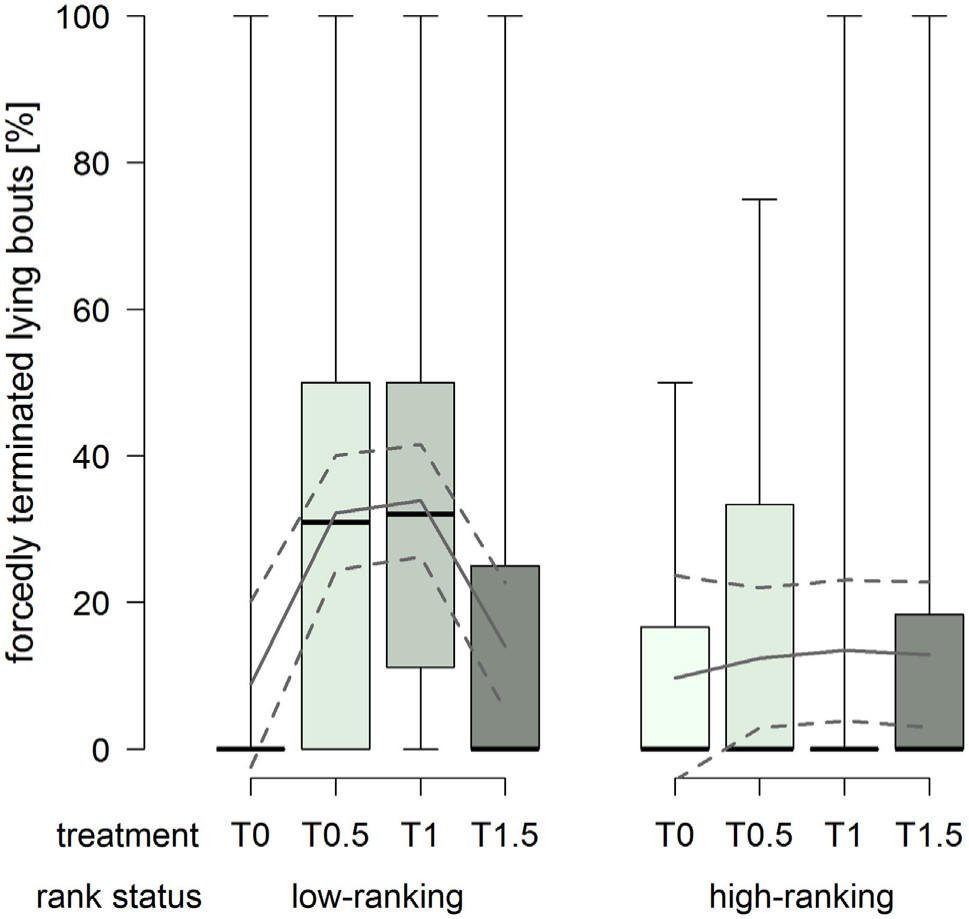
**Proportion of forcedly terminated lying bouts in treatments T0, T0.5, T1, and T1.5 for the rank status “low-ranking” and “high-ranking.”** Boxplots show medians, interquartiles, and absolute ranges of data. In addition, model estimates (solid lines) and 95% confidence intervals (dashed lines) are shown.

The proportion of forcedly terminated lying bouts was constantly low in high-ranking horses, whereas low-ranking horses had considerably higher proportions in treatments T0.5 and T1 (treatment^2^ × rank status: *F*_1,76_ = 8.43, *p* = 0.005; Figure 6).

## Discussion

The lying behavior of the horses in the present study was influenced by the availability as well as the space allowance of the littered area. If only rubber mats were available, the total number of lying bouts and the total duration of recumbency were considerably lower in comparison to treatments providing litter and, further, the majority of horses was recumbent for less than 30 min per 24 h. If litter was provided, on the other hand, durations of recumbency on rubber mats as well as on firm ground were close to 0. These results evidently indicate an avoidance of rubber mats or firm ground for recumbency and show a clear preference for litter, i.e., a soft and deformable surface. Similarly, individually stabled horses preferred littered areas toward concrete floor when given the choice (38) and Muggenthaler et al. (57) found shorter durations of recumbency on rubber mats than on litter in group-housed horses with both materials equally available.

If litter was available, increasing dimensions of the littered area resulted in increased durations of recumbency. In accordance therewith, previous studies also found a positive correlation between the duration of recumbency and the space allowance of the littered area, i.e., a lower total duration of recumbency in group-housed horses with smaller space allowances of the littered area compared to group-housed horses with larger dimensions at their disposal (35, 37). With regard to the legally required minimal dimensions for the littered area in Switzerland (43, 44), a reduction in the dimensions of the littered area from treatment T1 to T0.5 caused a decrease in the durations of recumbency both in total and on litter as well as a higher probability for an individual to be recumbent for less than 30 min per 24 h on litter. In compliance with Dallaire (2), we assume that the limited availability of soft and deformable surface led to a shift from sleep in recumbent position (non-REM and REM sleep) to sleep in standing position (non-REM sleep only). On the other hand, the enlargement of the littered area from treatment T1 to T1.5 further increased the total duration of recumbency, although the effect was more pronounced in low-ranking than high-ranking horses, and clearly increased the duration of recumbency on litter regardless of the horses’ rank status. Accordingly, the enlarged dimensions of the littered area enabled the low-ranking horses to be recumbent equally long as the high-ranking horses and provided a greater opportunity for recumbency on litter instead of recumbency on rubber mats or firm ground. Nonetheless, certain individuals in the present study were observed recumbent on rubber mats or firm ground repeatedly and also for durations longer than 30 min per 24 h, a finding which has also been made in other studies (40, 57). This fact might be influenced by previous experiences of these individuals as Muggenthaler et al. (57) found differences between horses that had never been housed on rubber mats before and horses experienced with rubber mats; if half of the initially littered area was replaced by rubber mats, decreased duration of total recumbency was found in unexperienced horses but not in horses initially housed on only rubber mats. Consequently, a habituation to rubber mats in the long term cannot be ruled out. Although the horses in the present study had no recent experiences with rubber mats in the lying area, some of them might have experienced rubber mats in the lying area at an earlier time.

Further, horses performed relatively more lateral recumbency with increasing dimensions of the littered area. Recumbency in lateral position indicates complete relaxation (2, 35); however, the position is not essential for REM sleep (13, 16). Our findings could be explained simply by the possibility for the animals to stretch out without physical contact with other horses. Accordingly, individually stabled horses spent more time in lateral position if kept in a larger compared to smaller box stalls (58). Nonetheless, the model estimates of the proportion of lateral recumbency in the present study were much lower compared to the reported mean ranges of 15–30% in feral horses (3, 28) and 4–30% in group-housed horses (37, 40, 59, 60). If we had not transformed our data, the model estimates of the proportion of lateral recumbency would increase continuously from 6.99% (0.68%, 13.3%) in treatment T0 to 9.07% (2.84%, 15.3%) in T0.5, 11.14% (5.03%, 17.26%) in T1, and 13.22% (6.93%, 19.51%) in T1.5; and would therefore have met the mean ranges in the literature. However, the distribution of the proportion of lateral recumbency in our data was strongly skewed. Consequently, the untransformed model estimates should not be considered as typical averages of a group but rather show that large variations between the different individuals of a group exist.

The proportion of group members present in the experimental area at the moment of lying down also increased with increasing dimensions of the littered area. Recumbency is a synchronous behavior in many species (20) and, hence, studies found decreased synchronization of recumbency with reduced space allowance of the littered area in cattle (61–63), sheep (64), and goats (65). In horses, resting behavior is synchronized but simultaneous recumbency of all group members is rare as there is always at least one horse stand-resting or awake (4, 21, 33). Therefore, both standing and recumbent horses at the moment of lying down were counted and our findings indicate that increased dimensions of the littered area enable the groups to rest more synchronously.

The proportion of forcedly terminated lying bouts was affected by both the dimensions of the littered area and rank status. High-ranking horses were forced to stand up to a rather low extent regardless of the dimensions of the littered area. At the same time, low-ranking horses were forced to stand up to substantially higher proportions in treatments T0.5 and T1. The low proportion in treatment T0 can be explained by the generally low occurrences of recumbency in this treatment. On the other hand, the increased proportions in treatments T0.5 and T1 indicate that low-ranking horses retreated from other, potentially higher ranking, group members. Therefore, dimensions of the littered area as provided in treatment T1.5 appeared to be necessary in order to allow low-ranking individuals to terminate their lying bouts self-determinedly to a similar extent as high-ranking horses. Fader (35) also observed resting behavior (standing, sternal and lateral recumbency) in 10 groups and found an extent of 5.6–53.1% forcedly terminated bouts with no apparent effect of the space allowance of the littered area, but rank was inversely proportional to the number of forcedly terminated bouts.

In agreement with our hypothesis, the lying behavior of the horses in the present study was further affected by rank status. This finding is consistent with several studies on group-housed horses. The total duration of recumbency was also found to be positively correlated with rank (35, 37, 39, 40, 66). Regarding recumbency in lateral position, Fader (35) observed that low-ranking horses rarely occupy this position in comparison to high- and medium-ranking horses. However, the author considered three rank statuses, which may explain that no effect was found in the present study. Contradictory findings have been made regarding the total number of lying bouts as Baumgartner (40) observed fewer lying bouts in low-ranking horses, Zeitler-Feicht and Prantner (37) did not find any differences, and the present results showed more lying bouts in low-ranking horses. However, Baumgartner (40) also found that low-ranking horses were lying less frequently in the lying area and—if lying in the lying area—less frequently on litter and more often on rubber mats than high-ranking individuals; a result which was found likewise in the present study as low-ranking horses generally had higher numbers of lying bouts on rubber mats than high-ranking.

Overall, for treatments providing litter, both the number of lying bouts and the duration of recumbency in the present study were generally in agreement with the literature on group-housed horses (35, 37, 40, 57, 67). However, large individual variations were observed within the 38 horses and among the 8 groups; a result which is also in line with other studies (13, 14, 35, 40, 68, 69). A number of individuals showed durations of recumbency of less than 30 min per 24 h not only with the minimal dimensions according to the Swiss animal welfare legislation (43, 44) but also with 1.5× minimal dimensions. Consequently, neither the minimal nor the enlarged dimensions in this study were sufficient in order for every single individual to perform the minimal duration of recumbency that is assumed to be essential for horses (12, 13, 27).

Rapid eye movement sleep deficiency because of recumbent sleep deprivation has not been sufficiently investigated in horses and research on possible implications or timeframe until occurrence of symptoms is missing. Nonetheless, REM sleep deficiency potentially results in an impairment of welfare and health, as reported in horses (4, 19, 21, 23–25), cattle (22), cats (70), mice (71), rats (72–74), and humans (75, 76). Additional research is therefore needed to understand if the desired state of undisturbed lying behavior for all members of a given group can be achieved by a further enlarged space allowance of the littered area. Moreover, factors of the stable design (i.e., geometry of the room or disposition of doors) and structuring of the lying area (i.e., partitioning walls or structural elements) should also be considered, as these may offer retreat opportunities to low-ranking horses (59, 69), and therefore facilitate lying behavior at a given space allowance of the littered area.

## Conclusion

In the present study, horses showed a clear preference for recumbency on litter, indicating a strong bias toward a soft and deformable surface. Rubber mats were no adequate substitute for litter as they were only used for recumbency reluctantly when no litter was available. A beneficial effect of enlarged dimensions of the littered area was shown by increased durations of recumbency and decreased proportion of forcedly terminated lying bouts in low-ranking horses. Taking this into account, it became evident that the minimal dimensions for the littered area as implemented in the Swiss animal welfare legislation do not ensure undisturbed lying behavior for all members of a given group.

## Ethics Statement

Ethical approval for the implementation of the study was obtained from the Veterinary Office of the Canton of Vaud in Switzerland (VD 2835, Approval No. 25060). The approval required a daily protocol on the general well-being of each individual, including monitoring of feeding, locomotion, and social behavior, which was conducted by the farm and horse owners.

## Author Contributions

All the authors contributed to the conception or design of the work, drafting and revising, and final approval of the version to be published.

## Conflict of Interest Statement

The authors declare that the research was conducted in the absence of any commercial or financial relationships that could be construed as a potential conflict of interest.
